# Telemedicine platforms must be leveraged to strengthen rural health systems

**DOI:** 10.1002/jmrs.609

**Published:** 2022-08-03

**Authors:** Sabe Sabesan, Daniel Xing, James Gallo

**Affiliations:** ^1^ Department of Medical Oncology, Townsville Cancer Centre Townsville University Hospital Townsville Australia; ^2^ College of Medicine and Dentistry James Cook University Townsville Australia; ^3^ Department of Radiation Oncology Townsville Cancer Centre Townsville University Hospital Townsville Australia

## Abstract

Benefit of telehealth goes beyond providing consultations. Telehealth can be used to enhance rural workforce capabilities and scope of practice as part of strengthening rural health systems.

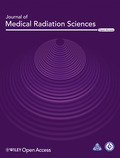

Telemedicine is a technology that has come of age, now it must be put to use to address inequities in cancer care. The principles and applications of telemedicine have undergone rapid evolution over the last two years globally.^1^, ^2^ Prior to the COVID‐19 pandemic, the utilisation of telemedicine was largely driven by local champions and lacked systematic uptake despite Government investment and incentives at both state and commonwealth levels. The pandemic and the resulting need for social distancing saw rapid and widespread adoption of telephone and telehealth services aided by Government funding, even for patients in metropolitan areas. Now is the time to leverage this momentum to embed telemedicine in our health systems.

Telemedicine has mainly been used for consultations and some clinical groups have used the platforms to build models of care and enhancing rural health systems.^3^ When specialist services are extended to rural communities using telehealth, local workforce capabilities and scope of practice also expand. Prior to the introduction of the teleoncology model in Mt Isa, all patients from this remote regional Queensland town had to travel thousands of kilometres to Townsville for medical oncology care. Now Townsville cancer centre provides a medical oncology service wholly via a teleoncology model; supported by a dedicated local workforce including rural doctors, nurses and allied health professionals.^3^ This increase in service capabilities, activities and scope of practice has necessitated the construction of a dedicated cancer centre locally as part of a regional cancer centre initiative. Not only do patients benefit from being cared for in their community but the community benefits from the decentralisation of infrastructure and service provision. The experience from early adopter sites will be invaluable to the strategic planning of rural health systems. So too will be the perspectives of the community leaders, families, nurses, allied health practitioners and doctors who implement telemedicine programs. Pow and colleagues make a valuable contribution to our understanding patient perspectives in their article related to telehealth enabled radiation oncology services in South Australia, but more work is still to be done.^4^

In many countries, including Australia and New Zealand, cancer centres have built on the existing telemedicine framework to provide remote chemotherapy and access to clinical trials.^2^, ^5^ The Telechemotherapy model sees rural nurses administer systemic therapy under the supervision and guidance of chemotherapy competent nurses, medical specialists and pharmacists. The Queensland Remote Chemotherapy Supervision (QReCS) model provides a governance framework to ensure the safety and quality of such services.^5^ After the introduction of the QReCS in Queensland, many rural sites including in New South Wales and Western Australia have also acquired the capabilities to administer selected chemotherapy regimens locally. Based on the success of the tele chemotherapy models, Australian governments have adopted the Australasian Teletrial Model (ATM) developed by the Clinical Oncology Society of Australia to enhance access to clinical trials closer to home.^2^ This national program aims to create an interconnected and networked clinical trial system across the country through regional clinical trials coordinating centres (RCCC), incentive grants, capacity building, as well as training and policy harmonisation. In the meantime, other clinical groups in cancer care have been promoting the telehealth model to achieve the same goals.

Pow and colleagues add further evidence to the literature that telehealth consultations can be acceptable to patients requiring radiation oncology care.^4^ They also show telehealth may reduce financial costs related to travel without compromising patient perceptions of quality, convenience and confidentiality. In fact, patients found that the quality of services and communication are similar to that of face‐to‐face care. Unfortunately, many patients continue to travel to larger centres for radiotherapy despite many rural and regional centres providing modern radiotherapy facilities in the public system or through private‐public partnerships. Is telehealth appropriate for initial consultations? Should telehealth be reserved for post‐treatment reviews or follow‐up consultations? Our own experiences from the medical oncology and radiation oncology departments within the Townsville Cancer Centre show that many initial consultations via telehealth can serve the purpose of full consultations provided the remote sites have the human resource and technological capabilities to provide suitable support.^6^ If remote sites do not have such capabilities, then it is important to triage and coordinate patient visits in order to minimise travel while critical investigations are pending. Telemedicine frameworks also provide the opportunity to strengthen intra and interprofessional ties between specialist centres and rural communities, to bolster case based learning and professional development and to improve utilisation rates of radiotherapy in rural and remote communities. Tele‐oncology also has the capacity to support the strong connection between Australia's first nations people, their communities and traditional lands by allowing treatment locally and possibly improving accessibility and the patient experience. These would all be worthy considerations when embedding “equity” into both public and private health care systems.

The results from this study strengthen the rationale to develop new models of care for radiation oncology leveraging the telemedicine platforms, as has already been shown possible by other clinical groups.^3^ For many radiation oncologists, remote planning has become common practice and provides the ability to work seamlessly between multiple geographically dispersed centres. It is likely that post treatment reviews and reviews of side effects can be conducted safely via telehealth for rural patients and that this may allow services greater agility and timeliness than conducting these visits face to face. We believe that some aspects of radiation trials can also be offered closer to home using the teletrial model including enrolment screening and consenting, selected treatment plans (where radiation treatment facility exists), post‐treatment follow up, patient reported quality of life measurement and medication adjustment. These approaches not only help create networks among healthcare professionals but also empower rural and remote communities. It is important to acknowledge that there are certain clinical scenarios in which examination by a specialised clinician is indispensable and where telehealth consultations may not be appropriate; for example, determining clinical tumour response after treatment, or where imaging may be insensitive at detecting early recurrence.

We believe that the foundation is set, and the time is right for radiation oncology to continue to build rural oncology services by formally incorporating telehealth into its model of care. The future model for rural radiation oncology services should involve shared care with rural generalists and general practitioners, case‐based discussions and training on cancer care and the management of common side effects from radiotherapy. As one of the next steps, the experience of early adopters, data from the published literature and input from stakeholders such as speciality colleges and cancer working groups could form the basis for nationally endorsed guidelines and frameworks to ensure broader adoption of telehealth in a safe and sustainable manner.

## Conflict of interest

No Conflict of interest to report.
